# Auditing Preparedness for Vector Control Field Studies

**DOI:** 10.4269/ajtmh.19-0710

**Published:** 2020-01-27

**Authors:** C. Matilda (Tilly) Collins, M. Megan Quinlan

**Affiliations:** Centre for Environmental Policy, Imperial College London, Silwood Park Campus, Ascot, United Kingdom

## Abstract

The value of baseline entomological data to any future area-wide release campaign relies on the application of consistent methods to produce results comparable across different times and places in a stepwise progression to larger releases. Traditionally, standard operating procedures (SOPs) and operational plans support this consistency and, thus, the validity of emergent data. When release plans include transgenic mosquitoes for vector control or other novel beneficial insects, additional factors come into play such as biosafety permits, stakeholder acceptance, and ethics approval, which require even greater coordination and thoroughness. An audit approach was developed to verify the correct use of SOPs and appropriate performance of tasks during mosquito mark, release, recapture (MRR) studies. Audit questions matched SOPs, permit terms and conditions, and other key criteria, and can be used to support subsequent “spot check” verification by field teams. An external team of auditors, however, was found to be effective for initial checks in this example before the use of a transgenic strain of laboratory mosquitoes. We recommend similar approaches for field studies using release of novel beneficial insects, to ensure useful and valid data as an outcome and to support confidence in the rigor of the step-wise process.

## CONTEXT AND RELEVANCE

Well-executed field studies provide the best data possible for decision-making before moving to pilot release studies using insects themselves as the control agent.^1–3^ The quality of the studies and their data become particularly important as the paradigm for novel vector control moves beyond methods of research to product development.^4^ This conceptual shift from localized to population-level control, gives baseline, longer-term monitoring and/or specific bio-ecological studies substantial value to future area-wide release campaigns using genetic technologies aimed at vector control.^3,5–7^

Mark, release, recapture (MRR) studies are particularly useful in understanding bio-ecological features of a target population, and additionally how a laboratory-reared mosquito may perform in a natural environment.^8–10^ Although there is always some variability in data from such field studies of natural populations, improving reliability and conformity of methods employed enhances reliability and confidence in any results used in decisions.

Historically, field entomology (FE) studies may have allowed for flexibility in operational planning to respond to real-world conditions encountered. “Local adaptations” of method, timing, and technique to fit the constraints or objectives of the context, however, may fail to achieve terms and conditions of a permit and reduce data comparability between studies or sites within larger research initiatives.^7^ Here, we share part of our experiences of proceeding with more rigorous field studies that inform critical decisions regarding novel vector control with released mosquitoes; we hope for feedback on options to establish and demonstrate field study preparedness and reliability of data.

## AN EVOLVING CONTEXT

The discussion surrounding the move from laboratory to field studies of an innovative vector control method is developing fast.^11–13^ Gene drive strategies introduced through release of laboratory-reared mosquitoes or other vectors present additional questions, and this conversation is taking place in a public arena where many views are expressed.^14^

The role of field studies such as MRR in building knowledge for pilot releases is well established. When any genetically modified insect release is planned, additional, important factors come into play such as public perception of safety,^15,16^ stakeholder engagement,^17–19^ approvals from institutional ethics boards and biosafety committees,^20,21^ and preparation of regulatory dossiers aimed at gaining permit approval.^22^

Against a long background of FE studies aimed at understanding mosquito biology, managers are often unaccustomed to the level of additional paperwork and verification to demonstrate good practice or to justify moving forward with studies comprising a step-wise progression into field use. In recent years in projects such as Target Malaria, https://targetmalaria.org, there has been specification of FE guidance documents and the development of training to support these.^7^ This documentation is most effectively specified through collaborative process to include a diversity of experience, so that its stipulations are widely achievable and comprehensible. Research initiatives using genetic vector control involve, at each step, international teams and/or teams involving different expertise and background and facing different levels of national and institutional resources†. Clear evidence-sharing within and between teams is a critical factor to successful decision-making in such research collaborations.^23^ Guidance and training are vital components of such initiatives but may be insufficient to ensure longer term understanding of, and adherence to, such guidance. Furthermore, such research teams need to comprehensively document compliance for both public and funder confidence.

## THE AUDIT PRACTICE AND EXPERIENCE

We developed an audit approach to verify administrative readiness, correct use of standard operating procedures (SOPs)/technical guidance, and appropriate performance of tasks in a specific context; that of evaluating “preparedness” for field studies. This synthesized various translational practices to create a detailed audit checklist, divided by themes or areas of responsibility and on a readily accessible Microsoft Excel platform.

The audit sequence followed the plan for an MRR as an example of a field study that should be standard and consistent. The process started with administrative preparation, and then continued through production of mosquitoes for release, transport to the study site, the actual field study phase, and laboratory identification of recaptured specimens to wrap-up and feedback. We viewed this from the start as a tool for internal learning and management rather than the one forming evidence for regulators: a tool to support confidence within field research teams, laboratory collaborators, and funders.

The approach was applied in the first example in one disease-endemic partner country in the Target Malaria research consortium, in conjunction with a trial run of an MRR study using wild-type mosquitoes in advance of approval for any genetically modified strain. In this case, the audit topics, including the specific questions developed by the various subject specialists involved, were supplied in advance to the FE, stakeholder engagement, and operational teams being audited because of the formative nature of this approach.^24^ As the formality of the audit process was particularly unfamiliar for the field teams, being more well known in laboratory practice, we first undertook a supportive and formative pre-audit to identify elements where specific improvement was essential a priori. This also emphasized areas in which understanding of objectives or the reasons behind the context was important for promoting compliance. We allowed 3 months between pre-audit and audit to give time for improvements in practice and additional training, as needed.

The audit was then carried out during an MRR study using a non-transgenic laboratory strain, with the core process taking place over approximately 3 days. Later follow-up was based on review of subsequent study reports and community feedback elements. Each topic matched relevant guidance, SOPs, permit terms and conditions, and other key criteria (see Table 1 for topics and sample questions), and the evidence required was predetermined. For the audit itself, a mixed, external team of experienced auditors with specialist responsibilities, along with representatives from other teams on the pathway to audit, was found to be effective for both checks and further formative feedback. The auditors used the detailed checklist to indicate the level of practice observed for each topic or question and provided comments to supplement the yes/no/partial/or not applicable result. Each auditor noted on the audit form their evidence for the level assigned, including particular documents, records, or simple observations by the auditor. A follow-up report and response plan completed the audit process.

**Table 1:** Audit sections and topics with representative sample questions

Audit sections	Topic	Example question
Administrative preparation	Regulatory	Have the terms and conditions of the permission been met or responded to?
	Preparation	Have any attendant National Biosafety Authority (NBA) observers been briefed in the study?
	Ethics	Have the Institutional Biosafety Committee and the Institutional Ethics Committee given permission for the study?
	Organisation	Is there an available administration plan with contact details of participants?
	Emergencies	Is there a communication chain of command in the event of an emergency?
	SE/communications	Have the field team been briefed on the requirements of the permit, the biosafety measures required, and the contingency plan?
Insectary and production	Colony confirmation	Has the level of insecticide resistance in colony mosquitoes been evaluated and found comparable or less than field levels?
	Setup	Are there sufficient supplies for colony augmentation?
	Augmentation	Has a calendar for the activities required for augmentation been prepared?
	Marking	Have the mosquitoes been colour-marked and has this application been verified?
	Packing	Are a sufficient number of cages available to take to the field for releases?
	Record keeping	Chain of custody: Has the insectary logbook been signed to confirm packing, number of coolers, and exit?
Transport out	Transport	Are two (or more) vehicles available, fit, and equipped correctly for transport?
	SE/communications	Can the drivers or key vehicle staff explain this activity within the parameters of the project message policy?
	Vehicle	Is there a copy of the incident procedure (Contingency Plan) in each vehicle?
	Journey	Have the mosquitoes arrived in a good condition for the study?
	Record keeping	Chain of custody: Was the Transport Record signed to acknowledge arrival at site?
Field preparation	Field laboratory	Is the field laboratory fit for purpose and lockable?
	Field team	Has the welfare of the field team been well considered?
	Preparation	Is the village acceptance of this study current and verified?
	Organisation	Are the method-specific training records available for all team members?
Field activities	Release	Were the mosquitoes released at the stipulated time and place?
	Swarm capture (by sweep net)	Were the swarm sampling locations available to the field team in advance?
	Pyrethroid spray catch	Was the pyrethroid spray catch procedure in accordance with the Field Guidance?
	Mosquito sorting	Are the samples being accurately tracked and documented?
	Record keeping	Is the sample labeling procedure correct?
Transport back	Transport	Was the principal investigator/project manager kept informed of departure from field and arrival at the laboratory
	Communication	Were the village authorities informed of the completion of the field study?
	Vehicle	Are the cargo’s accompanying documents present and correct?
	Journey	Was any incident handled appropriately? (if there were any)
	Insectary	Did the unloading happen as required?
	Record keeping	Was the transport record signed to acknowledge arrival at the insectary?
Insectary and identification	Re-entry	Have the transport cages been appropriately cleaned for return to containment?
	Preparation	Have the staff members performing the identification of field samples appropriately trained in molecular techniques?
	Analyses	Are laboratory notebooks and methodologies being correctly completed during the analyses?
	Sample handling	Are samples being correctly labeled and stored?
	Record keeping	Are the data sheets being correctly completed, correctly saved, and backed-up?
Wrap-up	Management	Were the principal investigator and project manager contactable throughout this study?
	Regulatory	Has the relevant authority (NBA or Ministry of the Environment) been informed of the end of the study?
	Record keeping	Have the data sheets been submitted to the parent project within the time stipulated?
	SE	Has the summary of initial study been shared with the village and local staff?
	Field entomology	Has the monitoring plan been agreed and is it being followed?

SE = represents stakeholder engagement.

In this instance, the formative process of pre-audit and subsequent summative audit (in which substantial and convincing adherence to guidance was recorded) led to auditors reaching agreement with the on-site team and the central management of the research consortium to recommend proceeding to the next step when regulatory permits allowed. Following this experience, teams can continue to use the audit framework to support subsequent “spot check” internal verification. In larger consortia, teams could apply this structure to audit each other, which is useful when resources do not allow for full external auditor teams to visit on a routine basis.

## THE FIELD SITUATION

In many research institutions, field and insectary/laboratory teams are often overlapping and used on several projects. This creates additional challenges to compliance when different projects or objectives require different standards of consistency and rigor. Although an overarching Code of Practice for the field from one project can provide guidance on a wide range of factors, this is hard to apply across different funder requirements and would require broad institutional support and enforcement. We found that a field audit as described could verify the level of preparedness for many things easily lost from sight in both routine practice and under the pressures of additional preparation and documentation requirements. Such common sense issues as the driver having a mobile phone with credit and everyone carrying personal ID cards can be overlooked, which is where simple check lists come into play. The use of between-team auditing may have additional advantage as there are circumstances in which effective participation of certain external auditors may be limited by sensitivities to increased numbers of visitors on site, the language abilities of the auditors and security concerns in more remote areas.

## WHAT LIES AHEAD?

This is the beginning of a long path supporting confidence in exploring the potential of genetic vector control technologies. We are working toward full internal confidence before attempting to fulfill the anticipated requirements of regulatory frameworks that are not yet fully defined. In this example, we have briefly described the development of an audit process to take place before the field study, which is perceived as more important when using novel technologies. Although audit timing and coverage of topics may be worth further discussion, we recommend similar approaches for field studies that may face greater scrutiny than that has been the case historically. With an audited and more formalized path in place, field studies can provide trusted data and support both internal and external confidence in the process. Here, the improvements in performance of tasks at all stages of the study and the quality and consistency of supporting documentation seen between the first formative audit and later summative audit were convincing in indicating clear benefit to the preparation process. Demonstrable quality of process validated by such an audit is an important foundation to the next steps in delivering reliable proof-of-principle studies in the field for the emerging novel vector control strategies. We hope for an ongoing discussion that will inform, advise, and refine the process.

## Footnotes

†Similar objectives of regulatory compliance, enduring record keeping, and utility of studies were described for studies in containment facilities,^25^ and an internal audit process was used to evaluate readiness for proceeding with key steps such as applications for import of novel organisms.^26^
